# Anxiolytic-like effects of *Pseudospondias microcarpa* hydroethanolic leaf extract in zebrafish: Possible involvement of GABAergic and serotonergic pathways

**DOI:** 10.1007/s13659-023-00399-8

**Published:** 2023-10-04

**Authors:** Donatus Wewura Adongo, Charles Kwaku Benneh, Augustine Tandoh, Robert Peter Biney, Kennedy Kwami Edem Kukuia, Priscilla Kolibea Mante, Benjamin Kingsley Harley, David Oteng, Emmanuel Aduboffour Appiah, Ernest Cudjoe Anorbor, Eric Woode

**Affiliations:** 1 Department of Pharmacology and Toxicology, School of Pharmacy, University of Health and Allied Sciences, Ho, Ghana; 2 Department of Pharmacotherapeutics and Pharmacy Practice, School of Pharmacy and Pharmaceutical Sciences, University of Cape Coast, Cape Coast, Ghana; 3 Department of Medical Pharmacology, University of Ghana Medical School, College of Health Sciences, University of Ghana, Korle Bu, Accra, Ghana; 4 Department of Pharmacology, Faculty of Pharmacy and Pharmaceutical Sciences, College of Health Sciences, Kwame Nkrumah University of Science and Technology, Kumasi, Ghana; 5 Department of Pharmacognosy and Herbal Medicine, School of Pharmacy, University of Health and Allied Sciences, Ho, Ghana

**Keywords:** Anxiety disorders, *Pseudospondias microcarpa*, Zebrafish, Novel tank, Benzodiazepines

## Abstract

*Pseudospondias microcarpa* is used in ethnomedicine to manage central nervous system diseases. The hydroethanolic extract (PME) from the leaves of the plant has shown anxiolytic-like properties in mice anxiety models. However, its effects in chronic anxiety models and possible mechanism(s) of action were not studied. Therefore, the current study evaluated the anxiolytic-like mechanisms of PME in zebrafish models of anxiety. The zebrafish light dark test (LDT) and novel tank test (NTT) were employed to assess the anxiolytic-like effects of PME (0.1, 0.3, 1.0 mg mL^−1^), fluoxetine (3 × 10^−5^ mg mL^−1^) and diazepam (1.5 × 10^−7^ mg mL^−1^). The chronic unpredictable stress (CUS) test was used to further evaluate the extract’s anxiolytic-like properties. The potential mechanisms of anxiolytic action of the extract was evaluated after pre-treated with flumazenil, granisetron, methysergide, or pizotifen, all at 1 × 10^−3^ mg mL^−1^. The extract significantly decreased anxiety behaviours in the NT and LD tests. These observed effects of the extract were however counteracted by flumazenil, granisetron, methysergide and pizotifen pre-treatment. In addition, PME treatment significantly reversed CUS-induced anxiety behaviours in zebrafish. Results show that PME possesses anxiolytic-like effects possibly through interaction with serotonergic and gamma-aminobutyric acid mediated pathways.

**Graphical abstract:**

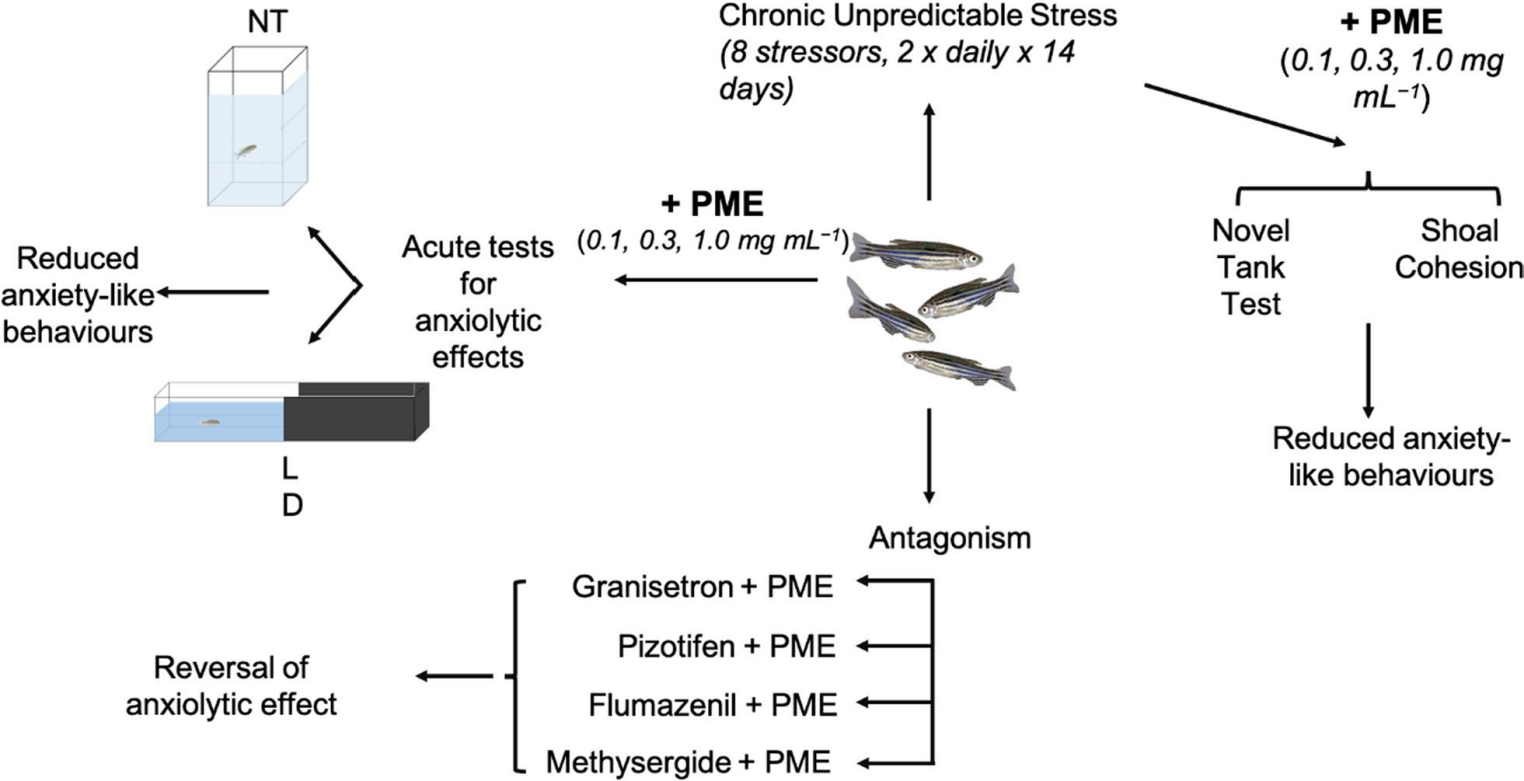

**Supplementary Information:**

The online version contains supplementary material available at 10.1007/s13659-023-00399-8.

## Introduction

Anxiety disorders typically depict physiological, psychological, and behavioural changes brought on by an actual or perceived threat to survival or well-being in humans or animals, and is often marked by an increase in nervousness, anticipation, hormonal and autonomic stimulation, as well as particular behavioural changes like feeding and exploration to escape [1]. The most prevalent psychiatric diseases globally are anxiety disorders, which also have a huge disease burden [2].

Antidepressants and benzodiazepines are two classes of medications that are frequently used to manage anxiety-related disorders. Although antidepressants including selective serotonin reuptake inhibitors (SSRIs) and serotonin norepinephrine reuptake inhibitors (SNRIs) are the preferred medications owing to their favourable benefit/risk ratio [2, 3], their use nevertheless results in sexual dysfunction and delayed anxiolytic effects [4–6]. Additionally, adverse effects could be more severe during the first 2 weeks. Initial jitters or an increase in symptoms regarding anxiety could happen, which could negatively affect patient adherence to their treatment regimen [2]. Benzodiazepines, unlike antidepressants, do not initially cause increased jitteriness and inability to sleep. However, they may cause CNS depression, leading to fatigue, drowsiness, slowed reaction times, declined cognitive function, dependence, and tolerance [2, 3].

Despite significant advancements, many people with anxiety disorders do not respond to pharmacological therapies in a satisfactory way [3]. This makes it necessary to identify and develop medications that are free of these tolerance and efficacy limitations [7]. In clinical studies, a number of medicinal plants including *Kava kava*, *Valeriana officinalis*, *Passiflora incarnata*, *Withania somnifera*, and *Hypericum perforatum* have revealed encouraging results in treating anxiety disorders [8]. Thus, research into medicinal plants may help in the identification and subsequent development of new agents for managing anxiety disorders.

In various regions of Africa, the plant *Pseudospondias microcarpa* is frequently used to treat diseases, including conditions of the central nervous system (CNS). The plant is alleged to sedate individuals who sleep or sit underneath it, hence local folks of the Akan tribe in Ghana popularly refer to it as *katawani* “close your eyes”. As a result, it is utilized in Ghana as a sedative and to treat common CNS diseases [9]. A previous investigation showed PME to have anxiolytic-like properties in rodent models of anxiety [10]. A different study also reported that PME exhibited similar effects to those of antidepressants probably through the 5-hydroxytryptamine (5-HT) pathway [11]. Additionally, the extract produced a fast-onset and long-lasting antidepressant-like activity in chronic animal models depicting human depression, improving cognitive function and reversing depression-induced anxiogenic behaviour [12, 13].

Although rodent models depicting human neuropsychiatric diseases have long been employed in the search for novel therapies, inefficient experimentation and likely high expenses remain barriers [14]. The zebrafish, an inexpensive, marine vertebrate species that shares a great deal of human genetic and physiological makeup, has in the last decade been recognized as a potent animal for modelling several CNS disorders in humans [15–18]. Additionally, approximately 82% of genes linked to human diseases have orthologues in the fully sequenced zebrafish genome [19].

According to a recent review, the zebrafish has been used in a number of research as an effective tool for finding natural therapies with possible anxiolytic benefits [20]. Although the anxiolytic effects of PME have been established in rodent models, its effects in chronic anxiety models and possible mechanism(s) of action are yet to be studied. Thus, this study explored the anxiolytic-like effects of PME in zebrafish models depicting acute and chronic anxiety states. In addition, the possible anxiolytic mechanism(s) were investigated.

## Results

### Analysis of PME with Fourier-transform infrared spectroscopy (FT-IR)

Over an IR band of 400–4000 cm^−1^, different functional groups were identified using FT-IR spectroscopy. In order to compare extracts afterwards, characteristic spectra in the region were employed as the fingerprint spectra. Additional file 1: Figure S1 and Table S1 show IR spectra and peak values respectively.

### Acute anxiolytic effects

#### Novel tank test (NTT)

**Fig. 1:**
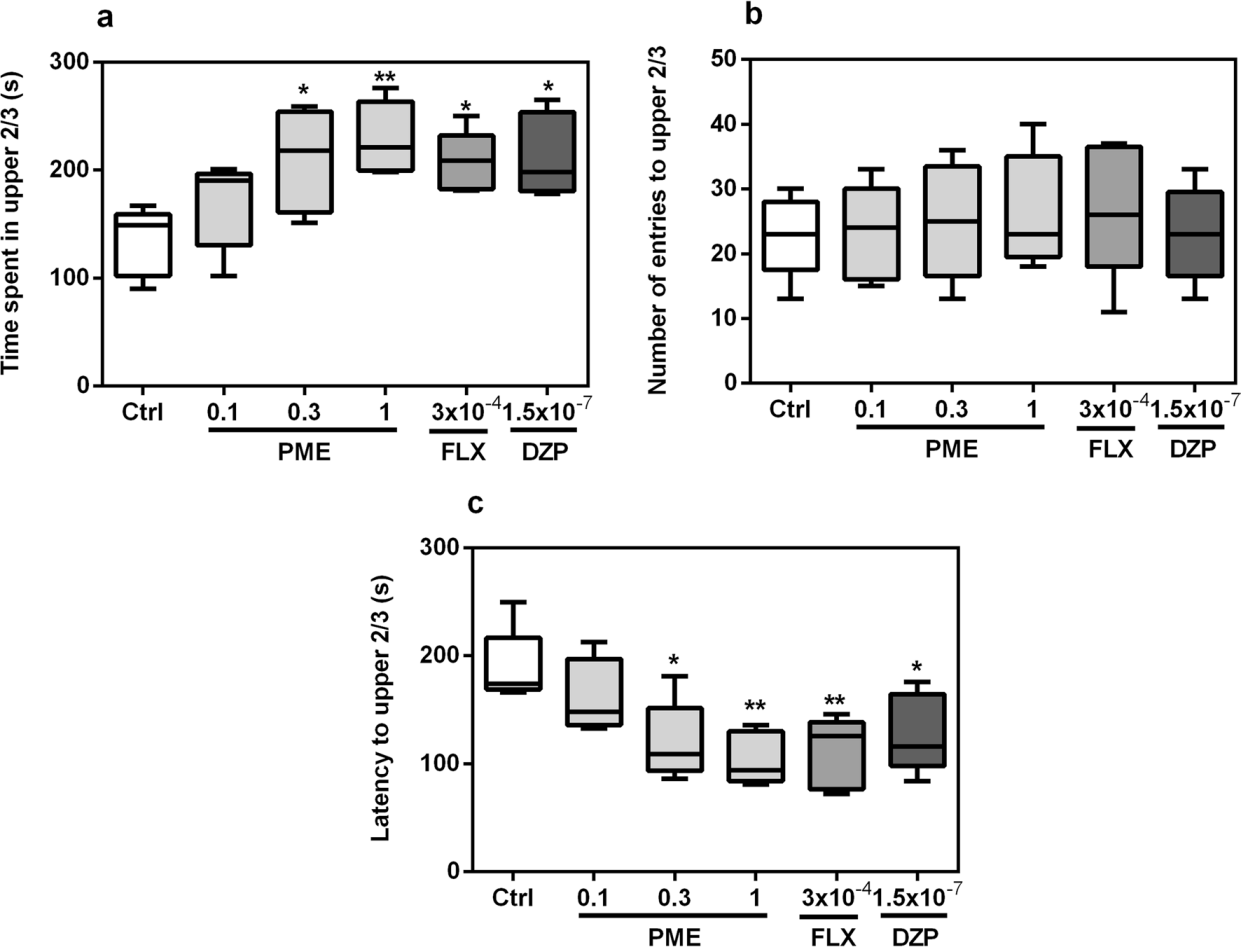
Effects of acute administration of PME, fluoxetine, and diazepam on time spent in upper 2/3 (**a**), number of entries to upper 2/3 (**b**) and latency to upper 2/3 (**c**) in the novel tank test. Data are expressed as group mean ± SEM (n = 5). Significantly different from control: *P < 0.05, **P < 0.01 (one-way ANOVA followed by Newman–Keuls post hoc test)

**Fig. 2:**
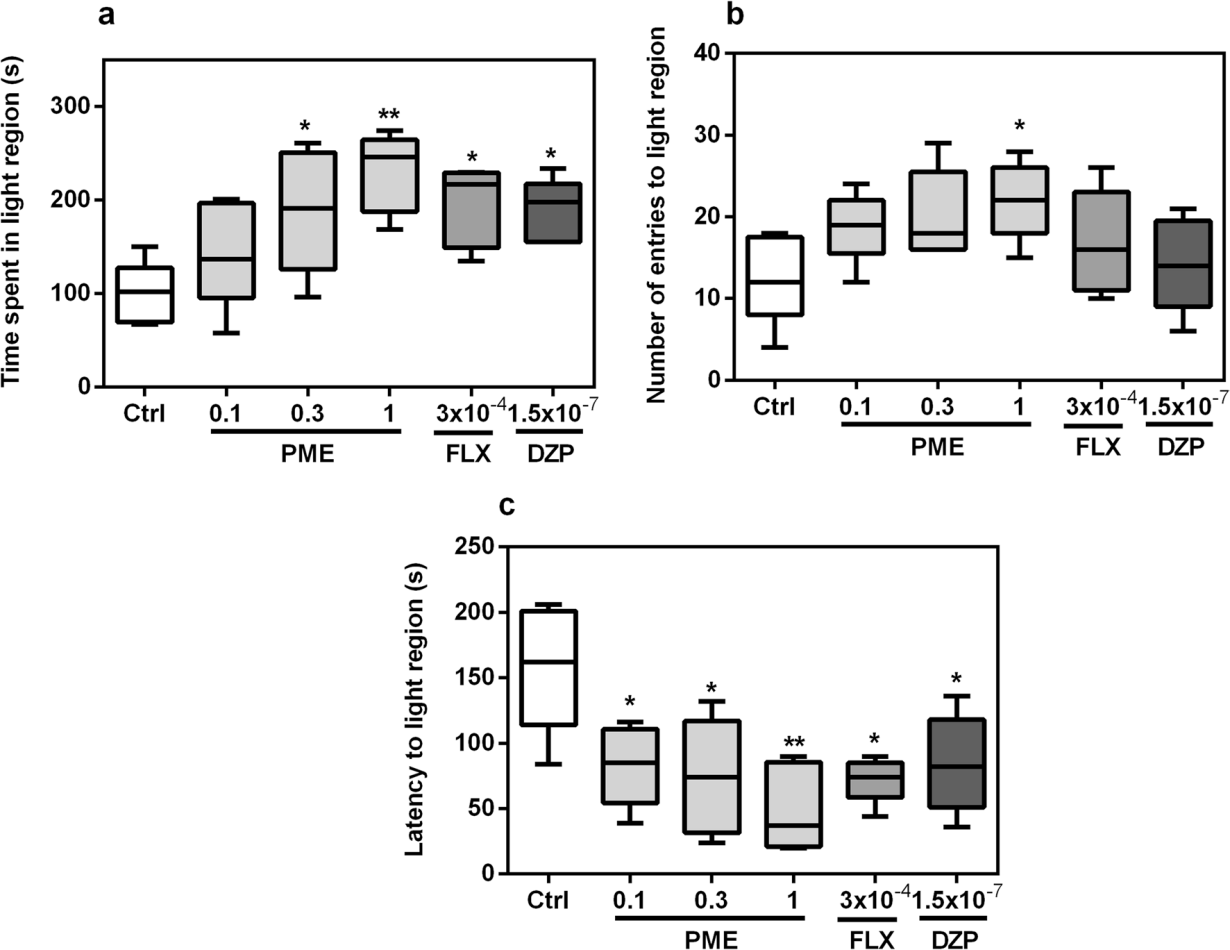
Effects of acute administration of PME, fluoxetine, and diazepam on time spent in light region (**a**), number of entries to light region (**b**) and latency to light region (**c**) in the novel tank test. Data are expressed as group mean ± SEM (n = 5). Significantly different from control: *P < 0.05, **P < 0.01 (one-way ANOVA followed by Newman–Keuls post hoc test)

The effects of acute treatment of PME, diazepam or fluoxetine on zebrafish behaviours in the NTT are shown in Fig. 1. The duration of fish in upper 2/3 compartments of the tank significantly increased following acute treatment with PME (*F*_5,20_ = 4.025, *P* = 0.0109). A post hoc analysis as seen in Fig. 1a showed significance at 0.3 mg mL^−1^ (*P* < 0.05) and 1 mg mL^−1^ (*P* < 0.01) for PME, and (*P* < 0.05) for both fluoxetine and diazepam. After acute treatment with PME, fluoxetine, or diazepam, latency to the upper 2/3 region (Fig. 2c) of the novel tank was significantly decreased (*F*_5,20_ = 4.866, *P* = 0.0045). However, neither PME nor the standard drugs had any statistically significant effects on entries (*F*_5,20_ = 0.249, *P* > 0.05) into the upper 2/3 region (Fig. 1b).

#### Light dark test (LDT)

Total time spent in the light region of the LD apparatus increased significantly after treatment with PME (*F*_5,20_ = 4.44, *P* = 0.0070). A post hoc analysis (Fig. 2a) showed significance at 0.3 mg mL^−1^ (*P* < 0.05) and 1 mg mL^−1^ (*P* < 0.01) for PME, and (*P* < 0.05) for both fluoxetine and diazepam. Latency to the light region (Fig. 2c) was also significantly reduced after acute administration of PME, fluoxetine, or diazepam (*F*_5,20_ = 4.625, *P* = 0.0058). Treatment with PME or the standard drugs did not affect the number of entries into the light region (*F*_5,20_ = 2.534, *P* = 0.0623), as shown in Fig. 2b. However, treatment with 1 mg mL^−1^ PME showed significance (*P* < 0.05).

### CUS

#### NTT

**Fig. 3:**
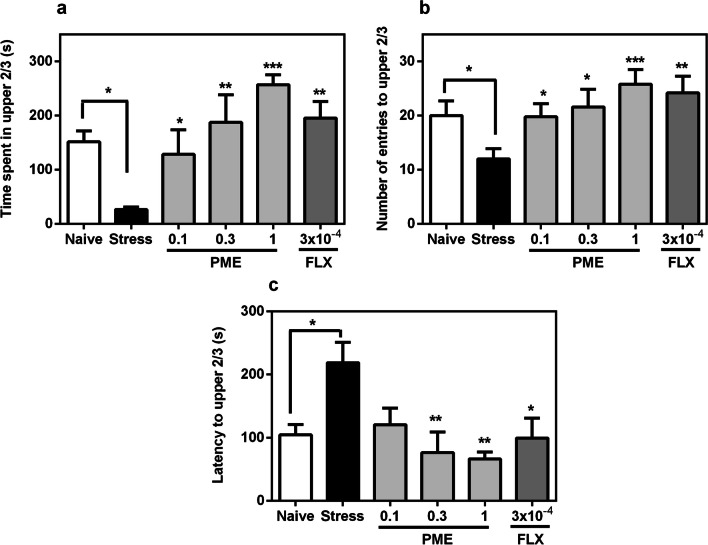
Effects of acute administration of PME and fluoxetine on time spent in light region (**a**), number of entries to light region (**b**) and latency to light region (**c**) in the novel tank test after the CUS procedure. Data are expressed as group mean ± SEM (n = 5). Significantly different from control: *P < 0.05, **P < 0.01, ***P < 0.001 (one-way ANOVA followed by Newman–Keuls post hoc test)

In comparison with the non-stressed group, zebrafish exposed to the CUS schedule displayed anxiety behaviours by showing increased latency to enter upper 2/3 region (*P* < 0.05) of the NT and spending less time (*P* < 0.05) in the same region (Fig. 3). With regards to the number of entries into the upper 2/3 region by stressed fish, a significant decrease (*P* < 0.05) was observed compared to the non-stressed group, indicating decreased locomotor activity. Treatment with PME or fluoxetine however significantly reversed these effects in the upper 2/3 regions as observed for time spent (*F*_5,20_ = 5.14, *P* = 0.0034; Fig. 3a), number of entries (*F*_5,20_ = 6.26, *P* = 0.0012; Fig. 3b), and latency (*F*_5,20_ = 4.18, *P* = 0.0092; Fig. 3c).

#### LDT

**Fig. 4:**
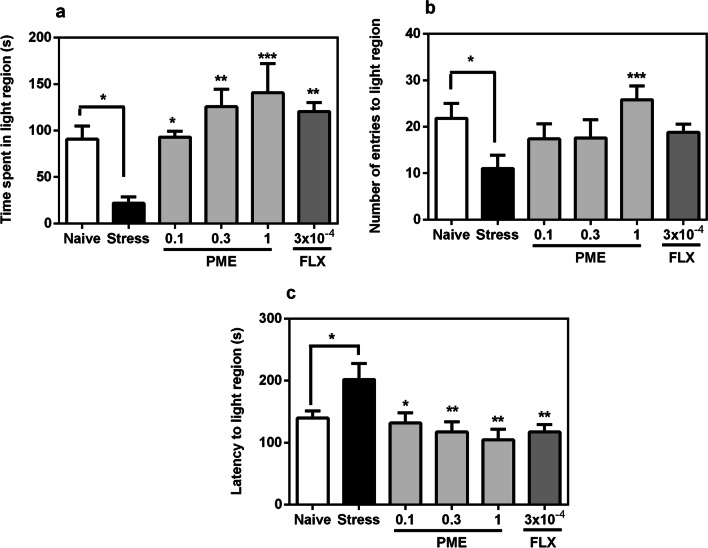
Effects of acute administration of PME and fluoxetine on time spent in light region (**a**), number of entries to light region (**b**), and latency to light region (**c**) in the light–dark test after the CUS procedure. Data are expressed as group mean ± SEM (n = 5). Significantly different from control: *P < 0.05, **P < 0.01, ***P < 0.001 (one-way ANOVA followed by Newman–Keuls post hoc test)

Chronic exposure of zebrafish to the CUS protocol resulted in reduced entries to the light compartment (*P* < 0.05) and decreased time spent in the same compartment (*P* < 0.05), while increasing the latency to entry (Fig. 4). However, PME or fluoxetine treatment demonstrated effects similar to anxiolytics by significantly increasing the time stressed zebrafish spent in the light region (*F*_5,20_ = 6.54, *P* = 0.0009; Fig. 4a) and decreasing latency to the light region (*F*_5,20_ = 5.06, *P* = 0.0037; Fig. 4c). The number of entries into the light compartment also increased significantly (*F*_5,20_ = 4.76, *P* = 0.0050; Fig. 4b), with PME at 1 mg mL^−1^ showing significance (*P* < 0.001).

#### Shoal cohesion

**Fig. 5:**
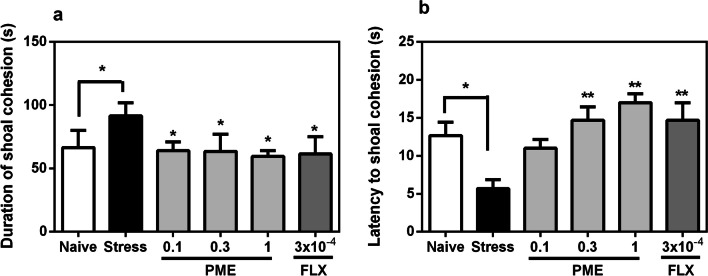
Effects of acute administration of PME and fluoxetine on shoaling cohesion duration region (**a**) and latency to shoal cohesion (**b**) after the CUS procedure. Data are expressed as group mean ± SEM (n = 3). Significantly different from control: *P < 0.05, **P < 0.01 (one-way ANOVA followed by Newman–Keuls post hoc test)

Stressed fish displayed noticeably altered shoal cohesion (Fig. 5). The duration of shoal cohesion in stressed fish increased considerably (*P* < 0.05), when compared to the naïve group. Additionally, a decrease in time taken to shoal cohesion in the CUS zebrafish (*P* < 0.05) was observed, indicating an anxiety state resulting from the CUS paradigm. However, acute treatment with the extract or fluoxetine decreased shoal cohesion duration (*F*_5,20_ = 9.37, *P* = 0.0141; Fig. 5a) and increased latency to shoal cohesion (*F*_5,20_ = 7.01, *P* = 0.0047; Fig. 5b) in stressed fish, indicating anxiolytic-like effect.

### Assessment of possible anxiolytic mechanisms

#### Involvement of the GABAergic system

**Fig. 6:**
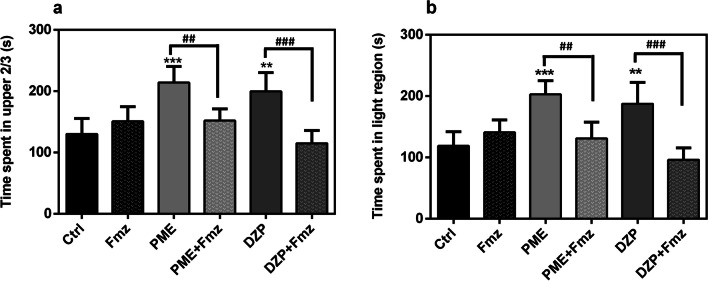
Effects of acute administration of PME (1 mg mL^−1^) and diazepam (1.5 × 10^−7^ mg mL^−1^) after pre-treatment with flumazenil (1 × 10^−3^ mg mL^−1^) on the time spent in upper 2/3 and light region of the novel tank test (**a**) and light–dark test (**b**) respectively. Data are expressed as group mean ± SEM (n = 5). Significantly different from control: **P < 0.01, ***P < 0.001 compared to control group; ^##^*P* < 0.01, ^###^*P* < 0.001 compared to group pre-treated with antagonist (one-way ANOVA followed by Newman–Keuls post hoc test)

As shown in Fig. 6, time spent in the upper sections of the NT and light compartment of the LD equipment were not significantly altered after immersion in 1 × 10^−3^ mg mL^−1^ flumazenil alone. Treatment with 1 mg mL^−1^ PME produced an anxiolytic-like effect similar to 1.5 × 10^−7^ mg mL^−1^ DZP, demonstrated by increased time spent in the upper 2/3 and light compartments in the NT and LD tests, respectively (*P* < 0.001 for PME and *P* < 0.01 for DZP in both tests). However, the observed anxiolytic-like effects of the extract in both experiments was reversed significantly with flumazenil pre-treatment (all at *P* < 0.01). Diazepam had similar effects as well.

#### Involvement of the serotonergic system

**Fig. 7:**
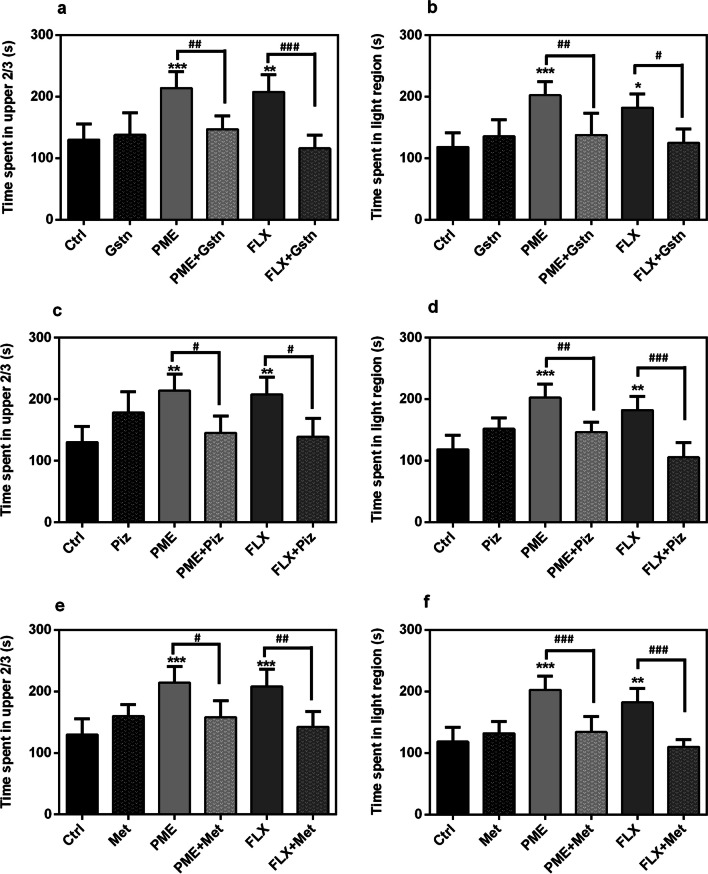
Effects of acute administration of granisetron (**a**, **b**), pizotifen (**c**, **d**) and methysergide (**e**, **f**) given alone or in combination with PME (1 mg mL^−1^) or FLX (3 × 10^−4^ mg mL^−1^) on the time spent in the upper 2/3 of the novel tank test and the light region of LDT. Data are expressed as group mean ± SEM (n = 5). Significant difference: *P < 0.05, **P < 0.01, ***P < 0.001 compared to control group; ^#^*P* < 0.05, ^##^*P* < 0.01, ^###^*P* < 0.001 compared to group pre-treated with antagonist (one-way ANOVA followed by Newman–Keuls post hoc test)

Figure 7 shows the effects of PME, fluoxetine or various serotonergic antagonists on fish behaviour in the NT and LD tests. In comparison to the control group, administration of granisetron, pizotifen, or methysergide (all at 1 × 10^−3^ mg mL^−1^) had no significant alteration on time spent in the upper 2/3 region of the NT. Similarly, the time spent in the light region did not alter significantly in the LDT.

Administration of PME or fluoxetine demonstrated anxiolytic-like effects by increasing the time spent in light region of the LD apparatus. However, the extract’s observed effect was blocked by pre-treatment with granisetron (*P* < 0.01; Fig. 7a), pizotifen (*P* < 0.05; Fig. 7c) or methysergide (*P* < 0.05; Fig. 7e). Fluoxetine showed comparable results: [granisetron (*P* < 0.001), pizotifen (*P* < 0.05), or methysergide (*P* < 0.01)].

Similar to effects in the NTT, administration of PME or fluoxetine increased significantly the upper 2/3 duration in the NTT. This was however reversed by pre-treatment with the various serotonergic antagonists: PME [granisetron (*P* < 0.01; Fig. 7b), pizotifen (*P* < 0.01; Fig. 7d) or methysergide (*P* < 0.001; Fig. 7f)], and fluoxetine [granisetron (*P* < 0.05), pizotifen (*P* < 0.001) or methysergide (*P* < 0.001)].

## Discussion and conclusion

In the zebrafish models of anxiety used in this investigation, administration of PME demonstrated anxiolytic-like activity comparable to that of fluoxetine and diazepam. In addition, the extract reversed anxiety state induced by the CUS paradigm confirming anxiolytic-like effects.

Most zebrafish behavioural models of anxiety were developed from rodent models, as these fish are often exposed to various stressors such as utilizing lit or dark areas, unfamiliar settings, and models of potential predators. Clinically effective anxiolytic drugs are used to validate these adjustments [20, 21]. This enables the investigation of possible anxiolytic effects of natural products.

The zebrafish instinctively seeks protection when placed in a novel environment, and the NTT is built on this behaviour. Zebrafish prefer to remain on the tank’s bottom until they feel safe enough to explore the whole tank [20, 22]. In order to evaluate anxiety in adult fish, the NTT generally measures a number of metrics including latency to explore the top, duration in the upper regions, entries to top, frequency of freezing episodes, and frequency of erratic engagements [23]. In this paradigm, increased irregular movements and freezing, together with a major reduction in exploration (increased latency to upper regions, decreased duration in upper regions, and fewer entries), are signs of elevated anxiety state and stress [24]. Drugs with anxiolytic effects, including benzodiazepines, antidepressants, and buspirone decrease the latency and increase exploratory behaviour in the upper regions [22, 25, 26]. The NTT was therefore used to evaluate acute anxiolytic effects of PME. The extract significantly decreased latency to enter the upper regions of the tank while increasing duration, indicating anxiolytic effects. Similar results were obtained for the anxiolytic drugs fluoxetine and diazepam. However, the total number of transitions to the upper regions of the NT did not significantly change with the doses of the extract used, eliminating any potential influence of locomotor activity on its anxiolytic effects. Comparable effects on locomotor function was observed for the standard anxiolytics used.

The light/dark test, which uses zebrafish’s natural aversion to highly lit places and their spontaneous exploratory behaviour in unfamiliar situations as an anxiety index, is another frequently employed behaviourally-validated test to evaluate anxiety in zebrafish [27]. Increased time spent in the dark by zebrafish (scototaxis) is a sign of an anxiety behaviour that is affected by both anxiogenic and anxiolytic drugs [28]. The extract significantly reversed scototaxis by increasing duration in the light compartment. Latency to light region was also decreased suggesting anxiolytic-like effects. The effects of the standard anxiolytics used in this test are consistent with previous reports where benzodiazepines and antidepressants produced anxiolytic effects [26, 29]. Locomotor activity in this test is measured by the frequency of crossings between the light and dark regions [29]. This parameter wasn’t decreased by acute treatment with the extract or standard drugs, suggestive of normal locomotor activity. The anxiolytic-like effects of the extract observed in the two models are quite consistent with previous studies in rodent models of anxiety [10, 13].

Numerous biological markers for central nervous system (CNS) drug testing have been discovered through the use of chronic stress-induced neuropsychiatric models in rodents in order to produce more effective therapies. However, using rodent models for CNS drug development is highly expensive [30]. Zebrafish are simple to handle and cost-effective for screening compounds as potential agents for treating CNS diseases, hence chronic models for anxiety and associated mood disorders have been designed. One of such is the CUS model which appears to be particularly effective in causing a pattern of behaviour of anxiety and other affective disorders in zebrafish [30, 31]. We therefore assessed behavioural effects of the extract including shoal cohesion in the CUS paradigm.

The behavioural studies demonstrated the effectiveness of the CUS paradigm in making fish extremely anxious, as shown by the decreased duration in light compartment, increased latency to light compartment and decreased transitions to light compartment in the LDT. Similar to the LDT, chronic exposure of fish to the CUS procedure also resulted in an anxiety state in the NTT. All these behaviours in the CUS fish reflect an anxiety state and are quite consistent with previous studies [26, 30, 32–34]. Numerous fish species have shown to engage in shoaling, a social and adaptive behaviour. Shoal cohesion, which is a pronounced propensity to form groups or shoals in zebrafish, is associated with feeding, predator defence, mating, and fear response [31]. Some studies have revealed a link between shoal cohesion and anxiety behaviour in zebrafish [30, 31]. In this study, exposure of fish to the CUS paradigm increased shoaling behaviour as observed by an increased shoal cohesion duration and decreased time to shoal formation, which is consistent with the study by [30]. Therefore, findings from the shoal cohesion indicate that after experiencing chronic unpredictable stress, zebrafish developed a phenotype associated with anxiety and other mood disorders. These behaviours were however reversed by PME and fluoxetine, further suggesting anxiolytic-like effects and confirming the observed effects in the acute anxiolytic studies. These results are quite similar to an earlier study we conducted where the extract reversed chronic unpredictable mild stress-induced anxiety in mice [13].

The neurotransmitter gamma-aminobutyric acid (GABA) is an important regulator of anxiety [35, 36], and zebrafish have shown to have a well-described GABAergic system [24, 37]. Similar to this, agents such as pentylenetetrazole that interfere with the GABAergic system in zebrafish induce convulsions. On the contrary, drugs that enhance GABAergic transmission such as diazepam attenuates convulsions [38]. Many natural compounds have GABA_A_ receptor-modulating activities because of the structural variety of GABA_A_ receptors [39]. Therefore, we used flumazenil to assess if the GABAergic system may be contributing to the anxiolytic-like effects of the extract. Flumazenil is a selective antagonist at the GABA_A_ receptor complex that has been shown to antagonize the sedative, anxiolytic, and anticonvulsant effects of benzodiazepines, making it a valuable agent for GABA_A_ receptor investigations [26, 40]. Pre-treatment with flumazenil reserved the extract’s anxiolytic-like effects, indicating a potential role of the GABA_A_ receptor complex. This finding is consistent with an earlier study which suggested that the GABAergic system may be implicated in the anticonvulsant activity of the extract [41].

The therapeutic benefits of anxiolytic medications in zebrafish have also been linked to the serotonergic system. [42]. According to studies, pharmacologically activating serotonin receptors reduces anxiety-like behaviour in zebrafish [20] and these days, such drugs are now the recommended first-line medications for treating anxiety disorders. In the present investigation, pre-treatment with the 5-HT receptor antagonists pizotifen, methysergide, and granisetron reversed the extract’s anxiolytic-like effects, suggesting the possible involvement of the serotonergic pathway. This could be as a result of the serotonin transporter being blocked, consequently increasing the concentration of 5-HT downstream and may possibly activate the 5-HT_1–3_ receptors directly or indirectly [26]. This is consistent with a prior study that demonstrated the extract’s antidepressant effects involved the serotonergic system [11]. Following pre-treatment with the antagonists, similar effects were seen for fluoxetine.

Overall, our results show that the hydroethanolic leave extract of *P. microcarpa* possess anxiolytic-like effects in acute and chronic zebrafish anxiety models and that this effect may be mediated via GABAergic and serotonergic systems.

## General experimental procedures

### Plant extraction

Leaves of *P. microcarpa* were harvested from the Kwame Nkrumah University of Science and Technology (KNUST) campus in Kumasi, Ghana (6° 40.626′ N, 1° 34.041′ W), and confirmed by Dr. George Sam of the Department of Herbal Medicine, KNUST. A voucher specimen with number KNUST/HM1/2013/L005 was then kept at the Faculty’s herbarium. After air-drying for a week, the leaves were pulverized into fine powder and cold macerated with 70% ethanol for three (3) days. A rotary evaporator with temperature set at 60 °C and under reduced pressure, was used to condense the filtrate into a brown syrupy substance. After a week of additional drying in a hot air oven at 50 °C, a yield (w/w) of 20.5% was obtained. The crude extract was labelled as PME.

### Fourier-transform infrared spectroscopy (FT-IR)

The spectrum two FT-IR spectrometer (PerkinElmer UATR Two) was used to conduct the FT-IR analysis in order to identify any possible functional groups that could possibly be present in the extract. The analysis was done over a range of 400–4000 cm^−1^ as this spectral region is unique for every compound or compound mixture.

### Chemicals and drugs

Diazepam (DZP) was obtained from Sigma-Aldrich, USA; fluoxetine (FLX) from Eli Lilly and Company Ltd., England; methysergide (Met) and pizotifen (Piz) were acquired from Novartis Pharmaceutical cooperation, Switzerland; granisetron (Gstn) from Corepharma LLC, England; and flumazenil (Fmz) from Roche Pharmaceutical Ltd., UK. Preparation of drug solutions was done with distilled water, and test compounds administered by immersing fish in 250 mL of the solution for 20 min. Based on preliminary tests and other studies, the doses of the agents employed in this investigation were selected [5, 26]. During the experiment, the extract concentrations used had no lethal or sedative effects on the zebrafish.

### Zebrafish

Aquarium Marshals Limited located in Accra supplied us with adult wild type zebrafish that were 3–5 cm long and 3 months old. Acclimatization of fish was done in 20 L glass tanks filled with dechlorinated water kept at 23–25 °C and a pH of 7–8. To reduce cross contamination, each tank had a separate water inlet and outlet. Each housing tank was planted with *Cabomba aquatica* and covered with gravel to a height of about 2 cm to simulate their natural habitat. Fish were maintained under a 14 h light/10 h dark cycle with lights switched on at 9:00 a.m. Commercial fish flakes and high-protein pellets were alternately fed to adult fish twice daily. Before the investigations, fish spent 15 days acclimating to the laboratory environment.

### Acute anxiolytic effects

#### Novel tank test (NTT)

The method as outlined by Benneh et al. was utilized [26]. The behavioural apparatus was a glass tank (15 cm × 10 cm × 25 cm) divided into three horizontal segments of equal dimensions by lines on the exterior of the tank, and filled with water to 18 cm. Zebrafish were given an acute treatment by immersion with PME (0.1, 0.3, 1.0 mg mL^−1^), fluoxetine (3 × 10^−5^ mg mL^−1^), diazepam (1.5 × 10^−7^ mg mL^−1^) or distilled water (control) for 20 min prior to the experiment. Following a gentle introduction into the test tank, each zebrafish’s behaviour was recorded with a camcorder for 5 min. Video outputs were analysed with the public domain software JWatcher™ for time spent in upper 2/3, number of entries to upper 2/3 and latency to enter upper 2/3. Increased anxiety is indicated by an inclination for lowest section of the tank and decreased exploration of the upper levels. A longer delay to enter the upper 2/3 is also suggestive of anxiety behaviour.

#### Light dark test (LDT)

The preference for a brightly lit or dark environment was evaluated using the light–dark apparatus based on the procedure as previously described [26]. The device measured 50 cm × 10 cm × 10 cm with its length divided into two equal halves, with either black or white backgrounds. Before the experiment, zebrafish were treated by immersion in PME (0.1, 0.3, 1.0 mg mL^−1^), fluoxetine (3 × 10^−5^ mg mL^−1^), diazepam (1.5 × 10^−7^ mg mL^−1^) or distilled water for 20 min. Following a gentle introduction into the test tank, each zebrafish’s behaviour was recorded for 5 min and analysed for the following parameters in the light region; total time spent, latency, and number of entries. Anxiolytic effects are indicated by a greater inclination for the light region, and evaluated by a longer stay there and more entry into the region. Decreased latency to light region is also considered as an anxiolytic behaviour.

### Chronic unpredictable stress (CUS)

#### Stressor pattern

The CUS procedure was performed as previously reported [26, 34]. Thirty (30) zebrafish were exposed to the stressors listed in Table 1 twice daily for 14 days. Repeated tank change (RTC) involved transferring fish quickly between tanks six times; dorsal body exposure (DBE) involved reducing the water level in the home tank for 10 min to expose the dorsal body surface; restrain stress (RS) required gently placing fish for 10 min in a 10 mL test tube half-filled with system water; social isolation (SI) entailed housing individuals in 100 mL beakers for 60 min; for overcrowding (OC), 10 fish were crammed into a 250 mL beaker that was only halfway full of system water for an hour; heat stress (HS) involved raising the tank’s water temperature to 33 °C for 30 min; cold stress (CS) entailed lowering the water’s temperature to 23 °C for 30 min; and chasing stress (C) defined as groups of zebrafish continually chased with a capture net for 5 min. In order to prevent habituation to stressors, the time and order of stressors were changed every day throughout the entire duration of the stressor schedule. With the exception of heating and cooling stressors, temperature and aeration were regulated throughout the execution of each stressor. The non-stressed group was designated naive control and kept in the same laboratory during the stress period.

**Table 1:** Chronic unpredictable stress (CUS) stressor pattern

	Day 1	Day 2	Day 3	Day 4	Day 5	Day 6	Day 7
Week 1							
Morning	RTC	OC	RS	SI	DBE	RS	CS
Evening	DBE	C	HS	CS	C	HS	OC
Week 2							
Morning	C	OC	SI	HS	DBE	CS	HS
Evening	RTC	DBE	RS	RTC	C	SI	OC

*RTC* repeated tank change; *DBE* dorsal body exposure; *RS* restrain stress; *SI* social isolation; *OC* overcrowding; *HS* heat stress; *CS* cold stress; *C* chasing stress

#### Behavioural testing and analysis

The NT, LD, and shoal cohesion tests were carried out simultaneously to analyse the behaviours of the naive and stressed groups 24 h after the CUS procedure. Stressed fish were randomly grouped (n = 5/group) as follows: control, PME (0.1, 0.3, 1.0 mg mL^−1^), and fluoxetine (3 × 10^−5^ mg mL^−1^). Zebrafish were dosed by immersing them in the drug solutions for 20 min before testing in the LD and NT tests described above.

Using the procedure outlined by Chakravarty et al. [30], the shoaling response was also evaluated. Videos were captured for 5 min after zebrafish from each group (n = 3) were placed into the NT. Shoal cohesion was measured as the time all three zebrafish swam together in the same quadrant. Latency to shoal cohesion was also measured.

### Assessment of possible anxiolytic mechanisms

The NT and LD tests described above were also employed to evaluate the possible contribution of the GABAergic and serotonergic systems in the anxiolytic-like effect of PME. Based on results from earlier studies, doses of the different antagonists were selected [26].

#### Involvement of the GABAergic system

This experiment evaluated the extract’s anxiolytic-like effects on the GABA_A_ receptor. Briefly, zebrafish (5 per group) were given one of the following treatments for 20 min: system water, diazepam (1.5 × 10^−7^ mg mL^−1^), PME (1 mg mL^−1^), or flumazenil (1 × 10^−3^ mg mL^−1^), followed by behavioural assessment in the NT and LD tests. Parameters measured were time spent in light and upper 2/3 compartments in the LDT and NTT respectively.

In a separate experiment done on the same day, fish were immersed in 1 mg mL^−1^ PME or 1.5 × 10^−7^ mg mL^−1^ diazepam for 20 min after being exposed to 1 × 10^−3^ mg mL^−1^ flumazenil for 20 min. Immediately after treatments, zebrafish were individually placed in the NT and the LD equipment for behavioural assessments.

#### Involvement of the serotonergic system

Serotonergic antagonists for the following receptors; 5-HT_1_ and 5-HT_2A/2C_ (pizotifen), 5-HT_2C/2B_ (methysergide) and 5-HT_3A/3B_ (granisetron) were utilized to evaluate the possible participation of the 5-HT system in the extract’s anxiolytic-like effects. Briefly, zebrafish (5 per group) were given one of the following treatments for 20 min: 3 × 10^−4^ mg mL^−1^ fluoxetine, 1 mg mL^−1^ PME, 1 × 10^−3^ mg mL^−1^ granisetron, 1 × 10^−3^ mg mL^−1^ pizotifen, 1 × 10^−3^ mg mL^−1^ methysergide or distilled water, followed by behavioural assessment in the NT and LD tests. Parameters measured were time spent in light and upper 2/3 compartments in the LDT and NTT respectively.

In another experiment, fish were immersed in 1 mg mL^−1^ PME or 3 × 10^−4^ mg mL^−1^ fluoxetine for 20 min after being exposed to 5-HT antagonists (all at 1 × 10^−3^ mg mL^−1^) for 20 min. Immediately after the various treatments, zebrafish were placed individually in the NT and the LD equipment for behavioural assessments.

### Statistical analysis

All data are displayed as mean ± SEM or box and whisker plots. The boxes’ lower and upper borders correspond to the 25th and 75th percentiles, and their extended arms, the 10th and 90th percentiles, respectively. One-way ANOVA was used to assess group differences, with the Newman–Keuls test used as a post hoc analysis. Statistical analysis was done using GraphPad Prism for Windows 5 (GraphPad Software, San Diego, USA) with significance set at *P* < 0.05.

### Supplementary Information

**Additional file 1: Figure S1.** Infrared spectrum of the hydroethanolic leaf extract of *P. microcarpa* (PME). **Table S1.** Peak table for IR spectra of the hydroethanolic leaf extract of *P. microcarpa* (PME).

## Data Availability

The datasets generated during and/or analyzed during the current study are available from the corresponding author on reasonable request.
