# Supplementary material for: Anxiolytic-like effects of Pseudospondias microcarpa hydroethanolic leaf extract in zebrafish: Possible involvement of GABAergic and serotonergic pathways
Source: Nat Prod Bioprospect. 2023 Oct 4;13(1):33. doi: 10.1007/s13659-023-00399-8 (PMC10547670; doi:10.1007/s13659-023-00399-8)
Supplement: Supplementary file 1 — Additional file 1: Figure S1. Infrared spectrum of the hydroethanolic leaf extract of P. microcarpa (PME). Table S1. Peak table for IR spectra of the hydroethanolic leaf extract of P. microcarpa (PME). [file 13659_2023_399_MOESM1_ESM.docx]

**Additional Material**

**
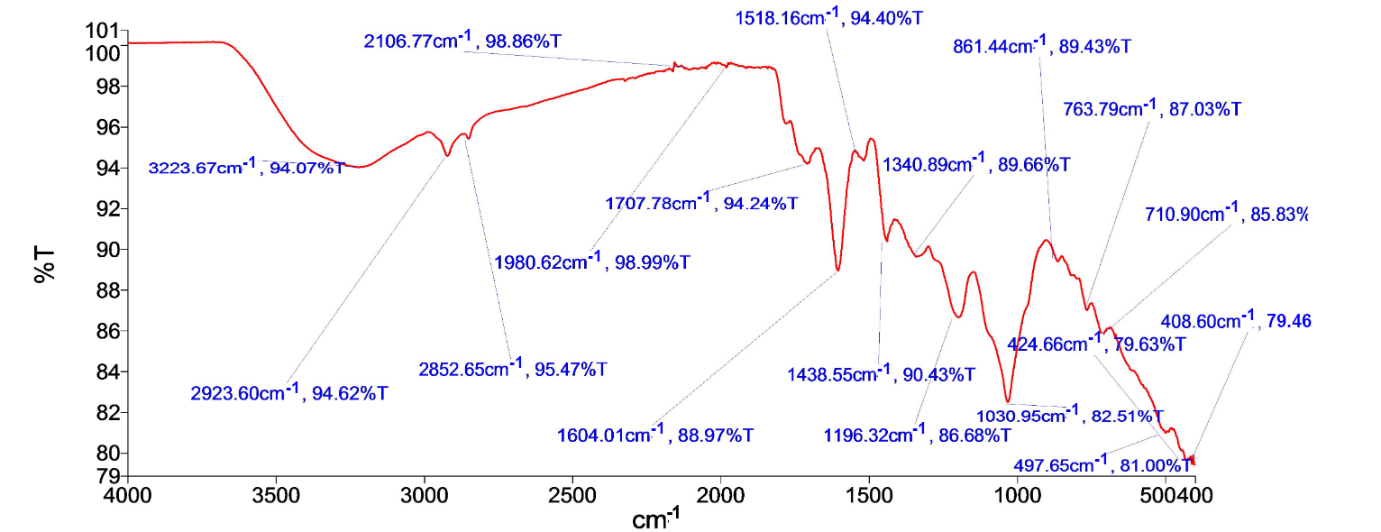
**

**Fig. S1** Infrared spectrum of the hydroethanolic leaf extract of *P.* *microcarpa* (PME)

**Table S1** Peak table for IR spectra of the hydroethanolic leaf extract of *P.* *microcarpa* (PME)

| **Peak** | **X(cm^-1^)** | **Y(%T)** | **Peak** | **X(cm^-1^)** | **Y(%T)** | **Peak** | **X(cm^-1^)** | **Y(%T)** | **Peak** | **X(cm^-1^)** | **Y(%T)** |
| --- | --- | --- | --- | --- | --- | --- | --- | --- | --- | --- | --- |
| **1** | 3223.67 | 94.07 | **2** | 2923.60 | 94.62 | **3** | 2852.65 | 95.47 | **4** | 2323.74 | 98.30 |
| **5** | 2162.45 | 98.77 | **6** | 2106.77 | 98.86 | **7** | 1980.62 | 98.99 | **8** | 1780.04 | 96.23 |
| **9** | 1707.78 | 94.24 | **10** | 1604.01 | 88.97 | **11** | 1518.16 | 94.40 | **12** | 1438.55 | 90.43 |
| **13** | 1340.89 | 89.66 | **14** | 1196.32 | 86.68 | **15** | 1030.95 | 82.51 |  |  |  |
